# Cerebral influx of Na^+^ and Cl^−^ as the osmotherapy-mediated rebound response in rats

**DOI:** 10.1186/s12987-018-0111-8

**Published:** 2018-09-25

**Authors:** Eva Kjer Oernbo, Kasper Lykke, Annette Buur Steffensen, Kathrin Töllner, Christina Kruuse, Martin Fredensborg Rath, Wolfgang Löscher, Nanna MacAulay

**Affiliations:** 1 Department of Neuroscience, University of Copenhagen, Copenhagen, Denmark; 2 Department of Pharmacology, Toxicology, and Pharmacy, University of Veterinary Medicine Hannover, Hannover, Germany; 3 Center for Systems Neuroscience, Hannover, Germany; 4 Neurovascular Research Unit, Department of Neurology, Herlev Gentofte Hospital, University of Copenhagen, Herlev, Copenhagen, Denmark; 5 Present Address: AJVaccines, Copenhagen, Denmark; 6 Department of Neuroscience, Faculty of Health and Medical Sciences, University of Copenhagen, Blegdamsvej 3, 2200 Copenhagen, Denmark

**Keywords:** Osmotherapy, Rebound effect, Brain edema, Brain barriers, Ion-transporting mechanisms

## Abstract

**Background:**

Cerebral edema can cause life-threatening increase in intracranial pressure. Besides surgical craniectomy performed in severe cases, osmotherapy may be employed to lower the intracranial pressure by osmotic extraction of cerebral fluid upon intravenous infusion of mannitol or NaCl. A so-called rebound effect can, however, hinder continuous reduction in cerebral fluid by yet unresolved mechanisms.

**Methods:**

We determined the brain water and electrolyte content in healthy rats treated with osmotherapy. Osmotherapy (elevated plasma osmolarity) was mediated by intraperitoneal injection of NaCl or mannitol with inclusion of pharmacological inhibitors of selected ion-transporters present at the capillary lumen or choroidal membranes. Brain barrier integrity was determined by fluorescence detection following intravenous delivery of Na^+^-fluorescein.

**Results:**

NaCl was slightly more efficient than mannitol as an osmotic agent. The brain water loss was only ~ 60% of that predicted from ideal osmotic behavior, which could be accounted for by cerebral Na^+^ and Cl^−^ accumulation. This electrolyte accumulation represented the majority of the rebound response, which was unaffected by the employed pharmacological agents. The brain barriers remained intact during the elevated plasma osmolarity.

**Conclusions:**

A brain volume regulatory response occurs during osmotherapy, leading to the rebound response. This response involves brain accumulation of Na^+^ and Cl^−^ and takes place by unresolved molecular mechanisms that do not include the common ion-transporting mechanisms located in the capillary endothelium at the blood–brain barrier and in the choroid plexus epithelium at the blood–CSF barrier. Future identification of these ion-transporting routes could provide a pharmacological target to prevent the rebound effect associated with the widely used osmotherapy.

## Background

The ion and fluid homeostasis in the mammalian brain is tightly controlled to preserve the intracranial pressure (ICP) within a normal range. Cerebral edema, as occurring in pathologies such as traumatic brain injury and stroke, can cause the ICP to rise to life-threateningly high levels [1]. In severe cases, a decompressive craniectomy can be initiated to lower the ICP [2]. Alternatively, osmotherapy can be used to osmotically extract cerebral fluid into the blood circulation by intravenous (i.v.) infusion of mannitol or NaCl [3], although it remains disputed which of these osmotic agents is most efficient for brain water extraction. The initial target when applying osmotherapy is a plasma osmolarity up to 320 mOsm but depending on the clinical circumstances, this recommended value may be exceeded [1]. Osmotherapy induces an immediate loss of brain fluid, which can, however, be reduced or even reversed due to yet incompletely understood mechanisms; a phenomenon referred to as the rebound effect [4, 5]. The rebound effect has been suggested to arise from a compensatory accumulation of cerebral osmolytes, generating an osmotic gradient favoring fluid movement back into the brain particularly upon dilution of the plasma osmolarity by renal excretion and/or withdrawal of the osmotic agent [4, 5]. It remains uncertain to what extent brain ion accumulation participates in the rebound response, and if so, which molecular transporting mechanisms contribute to this volume regulatory response. The secretion of ions may take place at one or both of the two major interfaces between the brain and blood: the capillary endothelium forming the blood–brain barrier (BBB) and/or the cerebrospinal fluid (CSF)-secreting choroid plexus epithelium, which forms the blood–CSF barrier (BCSFB) [6, 7]. The capillary endothelium and the choroid plexus epithelium express several ion-transporting mechanisms, i.e. the Na^+^–K^+^–2Cl^−^ co-transporter 1 (NKCC1), the Na^+^–H^+^ anti-porter 1 (NHE1), Na^+^-coupled bicarbonate transporters (NBCs), and the amiloride-sensitive Na^+^ channel (ENaC) [8–10]. These transport mechanisms may be potential candidates for brain ion and water regulation, and could, as such, participate in electrolyte translocation from blood to brain during the elevated blood osmolarity resulting from osmotherapy treatment. Inhibition of a subset of these ion transporters has been associated with improved outcome in an experimental animal model of stroke [11, 12], which may indicate involvement of such transport mechanisms in brain ion and water dynamics. Here, we employed in vivo investigations of healthy non-edematous rats to obtain the brain volume regulatory response to increased plasma osmolarity in the absence of pathological events, such as stroke/haemorrhage, and investigate a putative role of a range of transport mechanisms in the brain volume regulatory gain of ions.

## Methods

### Animals

This study was performed in accordance with the European Community guidelines for the use of experimental animals using protocols approved either by the Lower Saxony State Office for Consumer Protection and Food Safety (Niedersachsen, Germany) or Supervisory Authority on Animal Testing (Danish Veterinary and Food Administration, Denmark). To avoid variation due to mixed gender, only female Sprague–Dawley rats were employed, aged 9–13 weeks (Taconic A/S, Lille Skensved, Denmark or Janvier Labs, Le Genest-Saint-Isle, France). Whether the present findings hold for male rats as well will require further studies in the future. Rats were housed in groups of 2–5 per cage (Tp III cages, 22 °C, 12:12 h light/dark cycle) with access to unlimited water and standard altromin rodent diet. The allocation of rats into the treatment groups was randomized, and all experiments were reported in compliance with the ARRIVE guidelines (Animal Research: Reporting in Vivo Experiments) [13].

### Brain water extraction by elevated plasma osmolarity

Rats were anesthetized using isoflurane inhalation mixed in O_2_ (1.5–5%, 1 l/min) and anaesthesia was maintained throughout the entire experiment. The body temperature was controlled to 37 °C using an electric heating pad (Harvard Apparatus, Holliston, MA, US) and monitored by a rectal probe during the entire procedure. To avoid systemic regulation of blood osmolytes upon hyperosmotic treatment, a functional nephrectomy was performed immediately prior to the initiation of the experiment in all animals except for naïve animals, which were not exposed to isosmolar- or hyperosmolar treatment but underwent anaesthesia induction shortly before decapitation, see Table 1 for grouping of experimental animals. In brief, laparotomy incision areas were treated with local analgesia [2–4 drops 2% tetracaine (Sigma-Aldrich, Brøndbyvester, Denmark, T7508) or xylocaine (1 mg/ml, AstraZeneca A/S, Copenhagen, Denmark, N01BB02)/bupivacaine (0.5 mg/ml, Amgros I/S, Copenhagen, Denmark, N01BB01) (both in 0.9% w/v NaCl)] prior to opening of the abdominal cavity either from the dorsal or the ventral side in the fully anesthetized animals. The renal artery and vein were ligated using non-absorbable suture. For rats given ventral incision, a catheter (for i.p. delivery, see below) was placed during suturing of the incision, while for rats with dorsal incisions, the smaller openings were closed with metal wound clamps immediately after i.p. delivery. The rats received a single i.p. bolus of a physiological NaCl solution (0.9% w/v NaCl) as an isosmolar control treatment, while an equiosmolar bolus of NaCl (1.17 g/kg, 1 M [14]) or mannitol (7.29 g/kg, dissolved in 0.9% w/v NaCl; 2 M) was given to elevate the plasma osmolarity to a similar extent. All solutions were heated to 37 °C and delivered as 2 ml/100 g body weight. We employed i.p. delivery of the osmotic agent as this delivery route gives similar plasma osmolarities as i.v. delivery [14]. For i.v. inhibitor experiments, a catheter was inserted into the tail vein and an inhibitor mixture containing bumetanide (10 mg/kg [11], Sigma-Aldrich, B3023), amiloride (6 mg/kg [15], Sigma-Aldrich, A7410), and methazolamide (20 mg/kg [16], Sigma-Aldrich, SML0720) or vehicle (specified below) was injected 5 min prior to i.p. treatment with isosmolar- or hyperosmolar NaCl, see Table 1 for grouping of experimental animals. Inhibitors were given in a mixture to minimize the number of rats used for experiments. While drug concentrations in the blood are difficult to assess due to unspecific binding to tissue and blood proteins, we estimate maximal blood concentrations of 0.4 mM for bumetanide and amiloride, and 1.2 mM for methazolamide based on estimated blood volume of 7% of the rat body weight (average: 233 g). In a few experiments, rats were given a triple inhibitor or vehicle dose into the tail vein. In this case, a bolus injection of inhibitors or vehicle was given 20 min and 5 min before and 15 min after delivery of isosmolar or hyperosmolar NaCl. In other experiments, rats were positioned in a stereotactic frame (Stoelting, Wood Dale, IL, US, 51500) and a micro drill (CircuitMedic, Haverhill, MA, US, 110-4102) employed to induce a burr hole in the skull (coordinates from bregma: 1.4 mm lateral, 0.8 mm posterior). A Hamilton syringe (G27, Agntho’s AB, Lidingö, Sweden, 2100521) filled with inhibitor mixture (bumetanide: 33 μM, amiloride and methazolamide: 167 μM, final ventricular concentrations estimated to be 20 and 100 μM [17–22]) or vehicle dissolved in equilibrated (95% O_2_/5% CO_2_) artificial CSF (aCSF) (120 mM NaCl, 2.5 mM KCl, 1.3 mM MgSO_4_ × 7 H_2_O, 1 mM NaH_2_PO_4_, 25 mM NaHCO_3_, 10 mM glucose × H_2_O, 2.5 mM CaCl_2_, pH 7.4 at 37 °C) was fastened to the stereotactic apparatus and introduced into the right lateral ventricle (4.7 mm ventral). Two min prior to isosmolar or hyperosmolar i.p. treatment, 6 μl inhibitor or vehicle solution was injected in 2 s (volume and rate adjusted to hit both lateral ventricles), see Table 1 for grouping of experimental animals. To maintain an optimal intraventricular inhibitor dose, inhibitor or vehicle solution was injected into the ventricular system every 15 min. All the experiments were terminated by decapitation of the animal 1 h after i.p. injection of osmotic agent or physiological saline. A 1 h treatment period was chosen according to the reported near stabilization of plasma osmolarity and brain volume within 30 min after a hyperosmolar challenge [14]. All inhibitor solutions were made freshly each day (some from frozen stock solutions). Bumetanide and methazolamide were dissolved in 0.1 M NaOH (pH adjusted with 0.1 M HCl to pH 11 and 9, respectively) and diluted to 10 mg/ml for injection into the tail vein, while amiloride was dissolved in heated water at 10 mg/ml. Inhibitors, which were introduced into the ventricular system, were dissolved in DMSO (final concentration of 0.2% in aCSF).

**Table 1:** Overview of experimental animal groups

Experiment	Label	Osmotic agent	Treatment	Delivery route	# rats
Brain water and ion quantification	Control	–	Vehicle	i.v.	9
		–	Inhibitors	i.v.	7
		–	Vehicle	i.c.v.	6
		–	Inhibitors	i.c.v.	6
	Osmotherapy	NaCl (i.p.)	Vehicle	i.v.	9
		NaCl (i.p.)	Inhibitors	i.v.	8
		NaCl (i.p.)	3× vehicle	i.v.	4
		NaCl (i.p.)	3× inhibitors	i.v.	4
		NaCl (i.p.)	Vehicle	i.c.v.	6
		NaCl (i.p.)	Inhibitors	i.c.v.	6
		Mannitol (i.p.)	–	–	6
	Naïve	–	–	–	3
Brain barrier permeability	Control	–	NaFl	i.v.	3
	Osmotherapy	NaCl (i.p.)	NaFl	i.v.	3
	Naïve	–	–	–	3
Monitoring of ICP		–	Evans blue	i.c.v.	3
Blood pressure measurement		–	Vehicle	i.v.	3
		–	Inhibitors	i.v.	3

i.p., intraperitoneal; i.v., intravenous; i.c.v., intra(cerebro)ventricular; 3×, triple doses; NaFl, Na^+^-fluorescein

### Brain water and electrolyte quantification

The brain was removed immediately after decapitation. The olfactory bulbs and medulla oblongata were discarded and the remaining brain tissue was placed in a pre-weighed porcelain evaporation beaker and weighed within minutes after isolation to reduce loss of brain water. Brain tissue was homogenized in the pre-weighed evaporation beaker using a steel pestle and dried at 100 °C for 3–4 days to a constant mass for determination of the brain water content. The dried brain tissue (75–130 mg) was extracted in 1 ml 0.75 M HNO_3_ on a horizontal shaker table for 3 days at room temperature (RT). The Cl^−^ content in the brain extracts was quantified by a colorimetric method using a QuantiChrom™ Chloride Assay Kit (MEDIBENA Life Science & Diagnostic Solution, Vienna, Austria), while the Na^+^ and K^+^ content was quantified using flame photometry (Instrument Laboratory 943, Bedford, MA, US).

### Plasma osmolarity and ion quantification

A heparin-coated tube (Jørgen Kruuse A/S, Langeskov, Denmark) was filled with pooled blood (venous and arterial) from the neck region upon decapitation of the rats. Blood samples were kept cold for maximal 4 h until centrifugation at 1300*g* for 10 min at RT. The plasma layer was collected and stored at − 20 °C. The plasma osmolarity was determined by a freezing point depression osmometer (Löser, Berlin, Germany), while the content of Na^+^, Cl^−^, urea and creatinine was measured using a RAPIDLab^®^ blood gas analyzer (Siemens, Münich, Germany) or flame photometer (Instrument Laboratory 943).

### Analysis of data

If assuming that the barriers between blood and brain behave as semipermeable membranes, i.e. permeable only to water but not to solutes, a new steady state in brain water content V_h_ (h; hyperosmolar, in ml/g dry weight) mediated by an elevated plasma osmolarity ${\text{C}}_{\text{osm}}^{\text{h}}$ (mOsm) can be given by Eq. 1 as described in [14], where V_i_ is brain water content (i; isosmolar, in ml/g dry weight) in rats with isosmolar plasma osmolarity ${\text{C}}_{\text{osm}}^{\text{i}}$ (mOsm).

1 $${\text{V}}_{\text{h}} = {\text{V}}_{\text{i}} \cdot \frac{{{\text{C}}_{\text{osm}}^{\text{i}} }}{{{\text{C}}_{\text{osm}}^{\text{h}} }}$$

If the brain water loss is less than predicted by Eq. 1, this will imply that the brain gains osmotically active solutes given that the plasma and brain water is in osmotic equilibrium. The predicted gain of electrolytes, ΔQ (mmol/kg dry weight), can then be given by

2 $$\Delta {\text{Q}} = {\text{V}}_{\text{h}} \cdot {\text{C}}_{\text{osm}}^{\text{h}} - {\text{V}}_{\text{i}} \cdot {\text{C}}_{\text{osm}}^{\text{i}}$$

### Brain barrier permeability

To assess the paracellular permeability of the brain barriers, anaesthetized rats were subjected to a functional nephrectomy. The experiments were initiated as above, after which a 4% Na^+^-fluorescein (Sigma-Aldrich, F63772) solution (2 ml/kg, 0.25 ml/min, dissolved in 0.9% w/v NaCl) was infused into the femoral vein through a catheter; Na^+^-fluorescein is a marker of paracellular permeability and has been used to identify paracellular BBB disruption by osmotic shock [23]. Five min hereafter, isosmolar or hyperosmolar NaCl was injected into the abdominal cavity as described above, see Table 1 for grouping of experimental animals. Rats were decapitated after 1 h, and the brains were removed immediately and frozen on crushed solid CO_2_. Coronal sections (12 μm) were cut in a cryostat and mounted on slides. Na^+^-fluorescein was visualized using an Axioplan 2 epifluoresence microscope (Carl Zeiss Vision, München-Halbergmoos, Germany) equipped with a Plan Neofluar and an AxioCam MR digital camera by use of the AxioVision 4.4 software (Carl Zeiss Vision, Birkerød, Denmark). Image acquisition was performed in a blinded fashion. Representative images were captured of brain regions comprising the neocortex, hippocampus, thalamus, and the lateral ventricle. The pineal gland was used as an internal positive control due to its lack of BBB [24]. Phase contrast images were included to visualize brain structures in transmitted light. Image processing (brightness and contrast) was performed using Adobe Photoshop (San Jose, CA, US).

### Monitoring of ICP

In order to monitor the ICP of the anaesthetized rats, a micro drill (1 mm bit) was applied to manually induce a burr hole into the skull until transparency was observed. The thin skull layer was gently ruptured using a 0.6 mm bit (without disruption of dura mater) after which a tweezer was employed to remove skull flakes. An epidural probe (Plastics One, Roanoke, VA, US, C313GS-5-3UP, 0 mm below pedestal) was gently placed onto dura mater, and fastened to the skull by cement (GC, Kortrijk, Belgium, Fuji I, 000136). ICP fluctuations were detected by PicoLog Recorder software (Pico Technology, Cambridgeshire, UK). To ensure proper probe insertion in the epidural space, the jugular vein was compressed before the beginning of each experiment and a raised ICP detected as a positive control. An Evans blue (Sigma-Aldrich, E2129) solution (0.003% w/v in 0.9% w/v NaCl) was infused into the right lateral ventricle (6 μl in total, 3 μl/s) using a Hamilton syringe, while ICP recordings were collected, see Table 1 for grouping of experimental animals. 10 min after intraventricular injections, rats were euthanized by decapitation. The brains were isolated and cerebral hemispheres separated to confirm intraventricular Evans blue staining.

### Blood pressure measurement

Female Sprague–Dawley rats (22–28 weeks) were anaesthetized with chloral hydrate (400 mg/kg, i.p.), see Table 1 for grouping of experimental animals. A catheter was inserted into the left femoral artery to measure the intra-arterial blood pressure (BioSys software, TSE Systems, Bad Homburg, Germany). The intra-arterial blood pressure was monitored until 1 h after i.v. injection of inhibitors (10 mg/kg bumetanide, 6 mg/kg amiloride and 20 mg/kg methazolamide) or vehicle, and experiments were terminated by decapitation of the rats.

### Statistical analysis

All data are given as mean values ± standard error of mean (SEM). To evaluate statistically significant differences between mean values of two groups, an unpaired two-tailed Student’s t-test was applied, while a one-way analysis of variance (ANOVA) followed by Dunnett’s or Tukey’s multiple comparisons post hoc test was applied to compare mean values of multiple groups. Comparison of two factors was evaluated by a two-way ANOVA followed by Tukey’s multiple comparisons post hoc test. p < 0.05 was considered statistically significant. All statistical analyses were performed in GraphPad Prism 7.0 (GraphPad Software, Inc., La Jolla, CA, US) and indicated in the respective figure legend.

## Results

### Osmotherapy caused cerebral water loss and influx of Na^+^ and Cl^−^

**Fig. 1:**
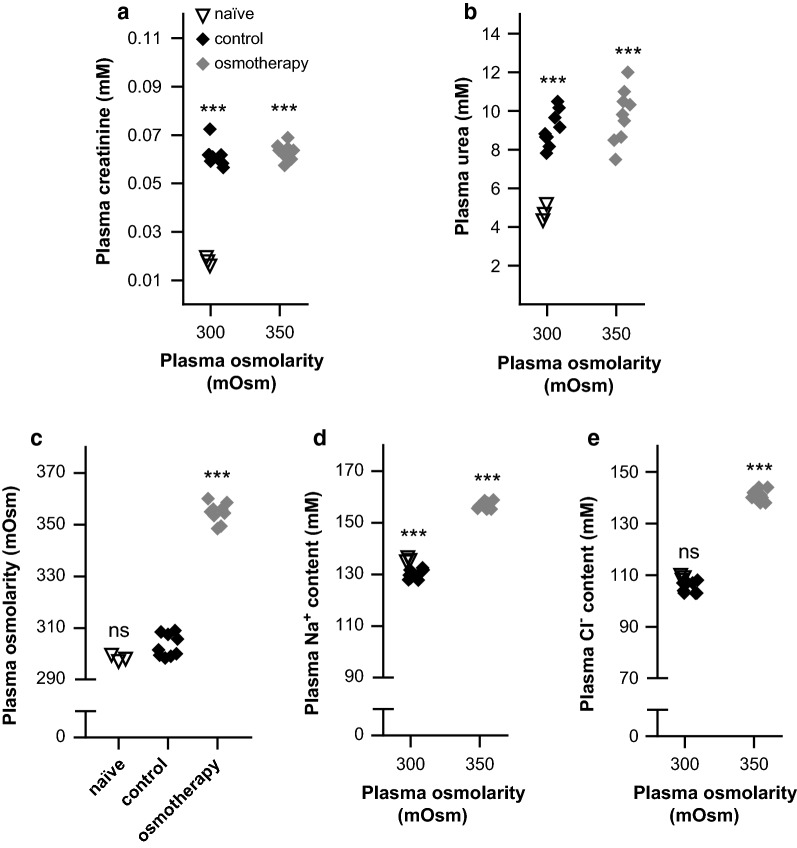
Plasma electrolyte concentrations in response to NaCl osmotherapy (elevated plasma osmolarity). A functional nephrectomy was performed in rats prior to i.p. treatment with isosmolar NaCl (control) or hyperosmolar NaCl (osmotherapy) and compared to non-operated naïve rats. **a** Plasma creatinine concentrations (in mM) in naïve rats (0.018 ± 0.001, n = 3), control rats (0.061 ± 0.002, n = 9), and osmotherapy-treated rats (0.063 ± 0.001, n = 9). **b** Plasma urea concentrations (in mM) in naïve rats (4.7 ± 0.2, n = 3), control rats (9.1 ± 0.3, n = 9), and rats exposed to osmotherapy (9.7 ± 0.5, n = 9). **c** Plasma osmolarity (in mOsm) of naïve rats (n = 3), control rats (n = 9), and rats exposed to osmotherapy (n = 9). **d**, **e** The plasma electrolyte concentrations (in mM) in naïve rats (135.6 ± 0.5 Na^+^ and 109.0 ± 0.6 Cl^−^, n = 3), control rats (130.0 ± 0.6 Na^+^ and 105.6 ± 0.7 Cl^−^, n = 9) and rats exposed to osmotherapy (156.5 ± 0.5 Na^+^ and 140.7 ± 0.8 Cl^−^, n = 9). Statistically significant differences were determined by a one-way ANOVA with Dunnett’s multiple comparisons post hoc test in **a**, **b** and Tukey’s multiple comparisons post hoc test in **c**–**e**. Asterisk above the scatter plots indicates statistical significance compared to naïve rats (**a**, **b**) or control rats (**c**–**e**). ***p < 0.001, *ns* not significant

To determine the effect of osmotherapy on the brain water and electrolyte content, we employed a rat in vivo model in which the plasma osmolarity was elevated by i.p. injection of NaCl (1.17 g/kg, 2 ml/100 g body weight). To isolate the effect of brain volume regulation, rats were functionally nephrectomized prior to the procedure, the success of which was evident from the increased plasma content of creatinine and urea in these animals compared to naïve rats, which had not undergone nephrectomy (Fig. 1a, b, see figure legend for values). The plasma osmolarity in the nephrectomized rats treated with isosmolar NaCl (303 ± 1 mOsm, n = 9, termed ‘control’ henceforward) was not significantly different from that of the naïve rats (298 ± 1 mOsm, n = 3, Fig. 1c), indicating that the extended experimental protocol in itself did not interfere with plasma osmolarity. Following a single bolus injection with hyperosmotic NaCl (termed ‘osmotherapy’ henceforward), the plasma osmolarity was increased to 355 ± 1 mOsm after 1 h (n = 9, p < 0.001, Fig. 1c), with an associated increase in the plasma content of Na^+^ and Cl^−^ (n = 9, p < 0.001, Fig. 1d, e, see figure legend for values).

**Fig. 2:**
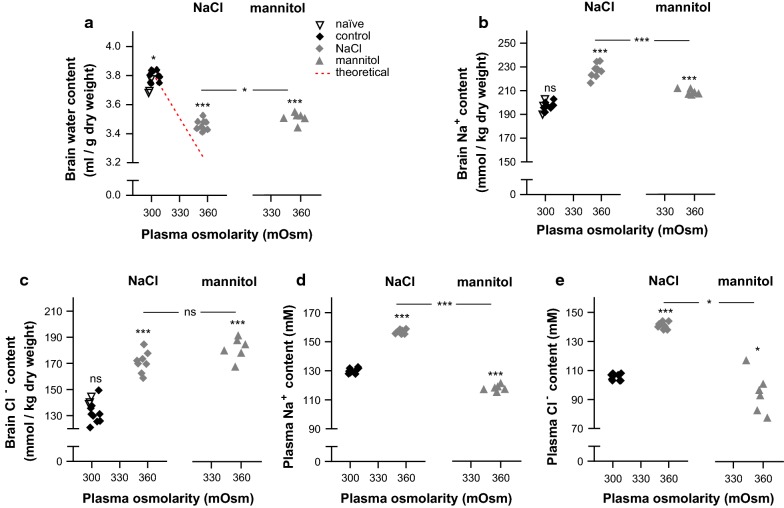
Osmotherapy-induced brain water loss and electrolyte gain. Rats were treated with isosmolar NaCl (control), hyperosmolar NaCl (osmotherapy, denoted NaCl in legend), or mannitol. **a** The brain water content (in ml/g dry weight) in naïve (n = 3) and control (n = 9) rats, and in rats exposed to osmotherapy in the form of NaCl (n = 9) or mannitol (n = 6). The theoretical brain water loss assuming ideal osmotic behavior (to 3.24 ml/g dry weight, calculated by Eq. 1) is illustrated as a dashed red line. **b**, **c** The brain electrolyte content (in mmol/kg dry weight) shown for naïve rats (197 ± 4 Na^+^ and 141 ± 2 Cl^−^, n = 3), control rats (197 ± 1 Na^+^ and 132 ± 3 Cl^−^, n = 9), and rats exposed to NaCl-mediated osmotherapy (227 ± 2 Na^+^ and 173 ± 3 Cl^−^, n = 9) or mannitol-mediated osmotherapy (209 ± 1 Na^+^ and 182 ± 3 Cl^−^, n = 6). **d**, **e** Plasma electrolyte concentrations (in mM) for rats exposed to mannitol-mediated osmotherapy (117.9 ± 0.8 Na^+^ and 94.9 ± 5.7 Cl^−^, n = 6). Values from control rats and rats exposed to NaCl-mediated osmotherapy are from Fig. 1d, e and included for comparison. To determine whether means of naïve, control, and NaCl were statistically different from each other, a one-way ANOVA with Tukey’s multiple comparisons post hoc test was performed. This statistical analysis was further performed to determine differences between means of control, NaCl, and mannitol groups [note; comparison of mannitol (no vehicle treatment) with either of the experimental NaCl groups (i.v. or intraventricular vehicle) provided similar results]. Asterisk above the scatter plot indicates statistical significance compared to control rats and asterisk within the lines indicates statistical significance between the indicated groups. *p < 0.05, ***p < 0.001

The brain water content of the naïve rats, which were not exposed to isosmolar or hyperosmolar treatment, (3.72 ± 0.03 ml/g dry weight, n = 3) was slightly lower than that of the control rats exposed to the isosmolar NaCl treatment (3.79 ± 0.01 ml/g dry weight, n = 9, p < 0.05, Fig. 2a), while osmotherapy caused a 9% reduction in the brain water content (to 3.46 ± 0.01, n = 9, p < 0.001, Fig. 2a). However, this reduction in brain water content amounted to only ~ 60% of that predicted from ideal osmotic behavior (calculated according to Eq. 1 and illustrated as a dashed red line in Fig. 2a), which indicates that volume regulation takes place. The osmotherapy-mediated reduction in the brain water loss was associated with an increase in brain electrolyte content, with a 15% increase in brain Na^+^ (p < 0.001, Fig. 2b) and a 31% increase in brain Cl^−^ (p < 0.001, Fig. 2c) (see figure legend for values). There was a minor 2% increase in the brain K^+^ content (control: 463 ± 2 mmol/kg dry weight vs. osmotherapy: 471 ± 2 mmol/kg dry weight, n = 9, p < 0.05). The brain Na^+^ and Cl^−^ content in control rats was not significantly different from that obtained in naïve rats, Fig. 2b, c, see figure legend for values. The total increase in osmolyte content represented by Na^+^, Cl^−^, and K^+^, ΔQ_observed_, amounted to 79 mmol/kg dry weight, which represents 104% of the predicted osmolyte gain, ΔQ_predicted_ = 76 mmol/kg dry weight (Eq. 2). The osmotherapy-mediated gain of brain Na^+^ and Cl^−^, and to a minor extent K^+^, can thereby account for the reduction in brain water loss observed 1 h after administration of the hyperosmolar challenge.

### NaCl is slightly more potent than mannitol in osmotherapy

To determine the potency of osmotherapy conducted with NaCl vs. mannitol, we performed a parallel experimental series with mannitol as the osmotic agent. The increased Na^+^ and Cl^−^ plasma concentration observed with NaCl infusion (as above, p < 0.001), was absent, and even slightly reversed compared to control rats upon i.p. delivery of mannitol (7.29 g/kg, 2 ml/100 g body weight) (p < 0.001 for Na^+^ and p < 0.05 for Cl^−^, Fig. 2d, e, see figure legend for values). Mannitol treatment yielded a plasma osmolarity (356 ± 3 mOsm, n = 6) similar to that obtained in rats treated with NaCl (355 ± 1 mOsm, n = 9, Fig. 1c, p = 0.71). Mannitol efficiently reduced the brain water content (to 3.51 ± 0.02 ml/g dry weight, p < 0.001), although slightly less effectively than NaCl (p < 0.05), Fig. 2a. Osmotherapy performed with mannitol increased the brain Na^+^ content by 6% (p < 0.001), which was less than with NaCl as the osmotic agent (15%, p < 0.001), Fig. 2b. The brain Cl^−^ content, in contrast, increased to a similar extent upon treatment with either of the osmolytes (31% with NaCl, p < 0.001 and 38% with mannitol, p < 0.001, Fig. 2c), which was also evident for the brain K^+^ content (p = 0.23; 2% with NaCl, n = 9, p < 0.01, and 3% with mannitol, n = 6, p < 0.001). Osmotherapy thus reduced the brain water content, but promoted brain electrolyte accumulation (predominantly in the form of Na^+^ and Cl^−^) irrespective of the osmotic agent employed, with NaCl being slightly more effective than mannitol for brain water extraction under our experimental conditions.

### Inhibitors of ion-transporting mechanisms at the blood-side membranes of the BBB capillary endothelium and choroid plexus had no effect on the brain water loss or electrolyte gain upon osmotherapy

**Fig. 3:**
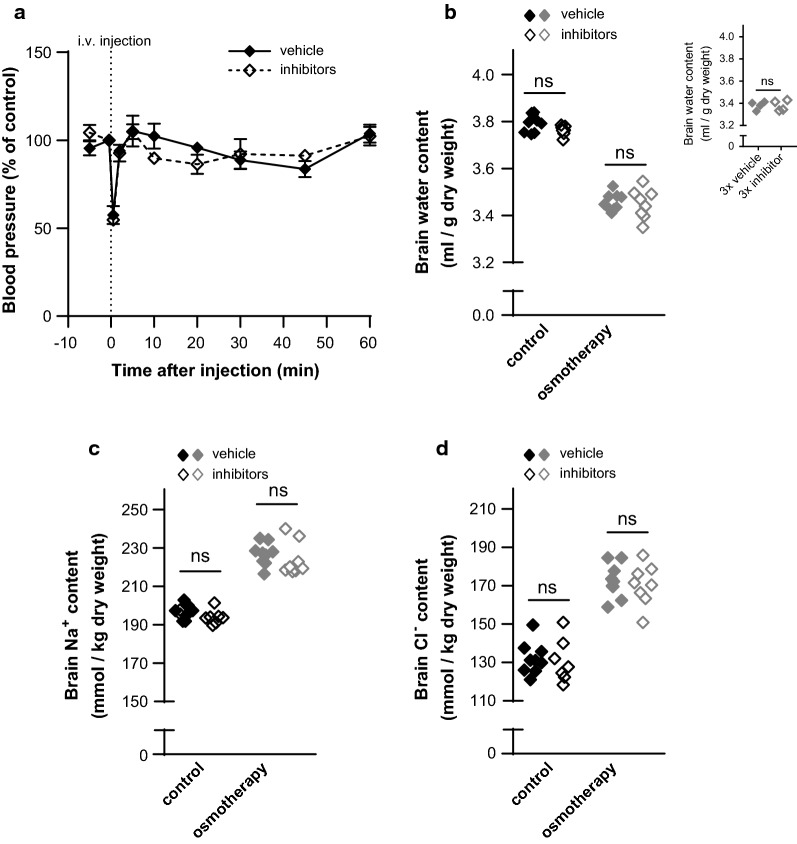
Inhibitors of ion-transporting mechanisms at the blood-side membranes do not affect water loss and electrolyte gain. **a** The arterial blood pressure was measured before and until 1 h after i.v. treatment with vehicle or inhibitors (10 mg/kg bumetanide, 6 mg/kg amiloride, and 20 mg/kg methazolamide). Values are given as the percentage of arterial blood pressure from the last control measurement (corresponding to 30 s before i.v. injection). The arterial blood pressure did not differ significantly from control measurements after 1 h (p > 0.90). The end arterial blood pressure was unchanged following inhibitor delivery, n = 3 of each, p > 0.90. **b** The brain water content was unaffected by i.v. inhibitor application in control rats [in (ml/g dry weight): vehicle: 3.79 ± 0.01 vs. inhibitors: 3.76 ± 0.01] and in rats subjected to NaCl-mediated osmotherapy (vehicle: 3.46 ± 0.01 vs. inhibitors: 3.45 ± 0.02), n = 7–9. Inset: Brain water content in osmotherapy-treated rats exposed to triple doses of vehicle (3.38 ± 0.02) or inhibitors (3.38 ± 0.02), n = 4 of each. **c** The brain Na^+^ content (in mmol/kg dry weight) in control rats (vehicle: 197 ± 1 vs. inhibitors: 194 ± 1) and in rats exposed to osmotherapy (vehicle: 227 ± 2 vs. inhibitors: 224 ± 3), n = 7–9. **d** The brain Cl^−^ content (in mmol/kg dry weight) in control rats (vehicle: 132 ± 3 vs. inhibitors: 131 ± 4) and in rats exposed to osmotherapy (vehicle: 173 ± 3 vs. inhibitors: 170 ± 4), n = 7–9. Vehicle values from control and osmotherapy-treated rats are from Fig. 2a–c and included for comparison. Statistically significant differences were determined by a two-way ANOVA with Tukey’s multiple comparisons post hoc test, except for values in the inset of **b**, which were analyzed using a two-tailed un-paired Student’s t-test. *ns* not significant

To identify the molecular mechanisms governing the hyperosmotic-induced brain ion accumulation and resulting volume regulation, the experimental regime from above (with NaCl as the osmotic agent) was repeated in rats during i.v. exposure to a mixture of inhibitors targeting a selection of ion-transporting mechanisms expressed in the BBB capillary endothelium and the blood-facing side of the choroid plexus. The diuretic compound bumetanide was applied for NKCC1 inhibition [25], amiloride to target NHE1 and ENaC [19], while the carbonic anhydrase inhibitor methazolamide [16] was applied to indirectly inhibit the NBCs. Importantly, these inhibitors did not demonstrate an effect on the arterial blood pressure of anaesthetized rats compared with vehicle (at 1 h endpoint, n = 3, Fig. 3a).

A single i.v. dose of inhibitors did not alter the plasma osmolarity compared to vehicle treatment in either control rats (vehicle: 303 ± 1 mOsm vs. inhibitors: 303 ± 2 mOsm, n = 7–9, p = 0.76) or osmotherapy-treated rats (vehicle: 355 ± 1 mOsm vs. inhibitors: 357 ± 2 mOsm, n = 8–9, p = 0.35). Delivery of inhibitors did not affect the brain water, Na^+^, and Cl^−^ content in control rats and failed to modulate the osmotherapy-induced changes in brain water, Na^+^, and Cl^−^ content, Fig. 3b–d. The K^+^ content was also unaffected by i.v. inhibitor application (in mmol/kg dry weight: control; vehicle: 463 ± 2 vs. inhibitors: 460 ± 2, osmotherapy; vehicle: 471 ± 2 vs. inhibitors: 474 ± 2, n = 7–9, p > 0.80). To increase the probability for the inhibitors to reach their targets in sufficient concentrations, we performed an additional experimental series with triple inhibitor application (20 min and 5 min prior to initiation of hyperosmotic treatment and 15 min after). These increased inhibitor doses did not affect the brain water content (Fig. 3b, inset). The unchanged electrolyte contents following inhibitor exposure aligns with the stable brain water content. These results suggest that NKCC1, NHE1, ENaC, and NBCs localized at the blood-facing side of the BBB capillary endothelium and the choroidal membrane are not the primary access routes for brain electrolyte entry during osmotherapy and therefore not the molecular mechanisms underlying brain volume regulation under these conditions.

### Osmotherapy-induced brain water loss and ion accumulation were unaffected by inhibitors of ion-transporting mechanisms at the CSF-facing choroidal membrane

**Fig. 4:**
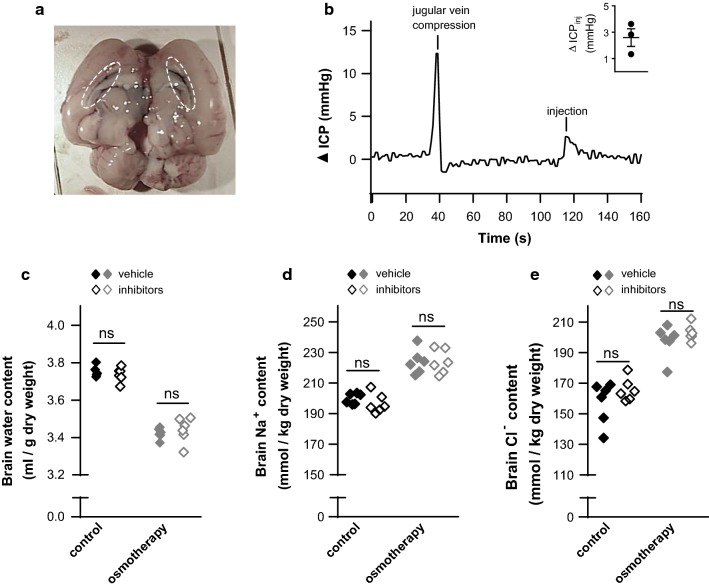
Inhibition of choroidal ion-transporting mechanisms does not affect brain water loss or electrolyte gain. **a** Representative image of brain hemispheres following Evans blue injection into the right lateral ventricle (stained lateral ventricles highlighted in dashed ovals), n = 3. **b** A representative epidural ICP trace with jugular vein compression included as a positive control. The inset shows mean ∆ICP ± SEM (mmHg) during intraventricular injection, n = 3. **c** Brain water content (in ml/g dry weight) of rats treated with intraventricular injections of vehicle or inhibitors prior to i.p. administration of isosmolar NaCl (control; vehicle: 3.75 ± 0.01 vs. inhibitors: 3.74 ± 0.02) or hyperosmolar NaCl (osmotherapy; vehicle: 3.42 ± 0.01 vs. inhibitors: 3.44 ± 0.03), n = 6 of each. **d** The brain Na^+^ content (in mmol/kg dry weight) in control rats treated with vehicle (200 ± 1) or inhibitors (197 ± 3) and in osmotherapy-treated rats exposed to vehicle (224 ± 3) or inhibitors (224 ± 3), n = 6 of each. **e** The brain Cl^−^ content (in mmol/kg dry weight) in control rats treated with vehicle (162 ± 3) or inhibitors (166 ± 3) and in osmotherapy-treated rats exposed to vehicle (198 ± 4) or inhibitors (203 ± 2), n = 6 of each. Statistical significant differences were determined by a two-way ANOVA with Tukey’s multiple comparisons post hoc test. *ns* not significant

Ion-transporting mechanisms localized at the other major interface; the ventricular side of the choroid plexus, may instead contribute to the volume regulatory gain of cerebral electrolytes upon administration of osmotherapy in the form of a hyperosmotic NaCl challenge. The select ion-transporting mechanisms expressed at the luminal membrane of the choroid plexus epithelium were targeted by injection of the inhibitor mixture (estimated ventricular concentrations of 20 μM bumetanide, 100 μM amiloride, and 100 μM methazolamide) directly into one of the lateral ventricles. Initially, the maximal inhibitor volume and infusion rate were chosen from two criteria: (1) both lateral ventricles should be exposed to inhibitors even though injections were given into only one of the lateral ventricles (verified with Evans blue, see Fig. 4a for a representative image) and (2) the ICP should remain fairly stable upon intraventricular inhibitor infusion (the ICP increased briefly to only a minor extent; 2.6 ± 0.7 mmHg, n = 3, Fig. 4b, with a brief compression of the jugular vein illustrated as a positive control). Vehicle or inhibitors were thus injected into the ventricular system of anesthetized rats prior to osmotherapy followed by another drug application every 15 min during the 1 h experimental time period to maintain a maximal targeting effect despite risk of wash-out by the high ventricular CSF flow rate [26]. The plasma osmolarity was similar in vehicle- and inhibitor-treated rats exposed to isosmolar NaCl solution (vehicle: 297 ± 2 mOsm vs. inhibitors: 298 ± 2 mOsm, n = 6, p = 0.94) and in rats subjected to osmotherapy (vehicle: 347 ± 1 mOsm vs. inhibitors: 347 ± 2 mOsm, n = 6, p = 0.87). Osmotherapy led to a reduction in the brain water content and to an increased Na^+^ (12%) and Cl^−^ (22%) content in the brain of vehicle-treated rats (n = 6, p < 0.001 for both, Fig. 4c–e), with an unaltered brain K^+^ content (in mmol/kg dry weight: control: 472 ± 3 vs. osmotherapy: 471 ± 4, n = 6, p > 0.90). Intraventricular inhibitor application had no effect on the brain water content in control rats or in osmotherapy-treated rats, Fig. 4c. The brain Na^+^ and Cl^−^ content in control- or osmotherapy-treated rats was, likewise, unaffected by inhibitor application into the lateral ventricles (n = 6, for all conditions, Fig. 4d, e), which was also seen for brain K^+^ content (in mmol/kg dry weight: control; vehicle: 472 ± 3 vs. inhibitors: 469 ± 4, osmotherapy; vehicle: 471 ± 4 vs. inhibitors: 472 ± 2, n = 6, p > 0.90 for both). These results suggest that osmotherapy-mediated brain electrolyte influx does not originate from increased activity of choroidal transporters (NKCC1, NHE1, NBCs, or ENaC) expressed at the luminal CSF-facing side of the membrane.

### The integrity of the brain barriers was preserved after osmotherapy treatment

**Fig. 5:**
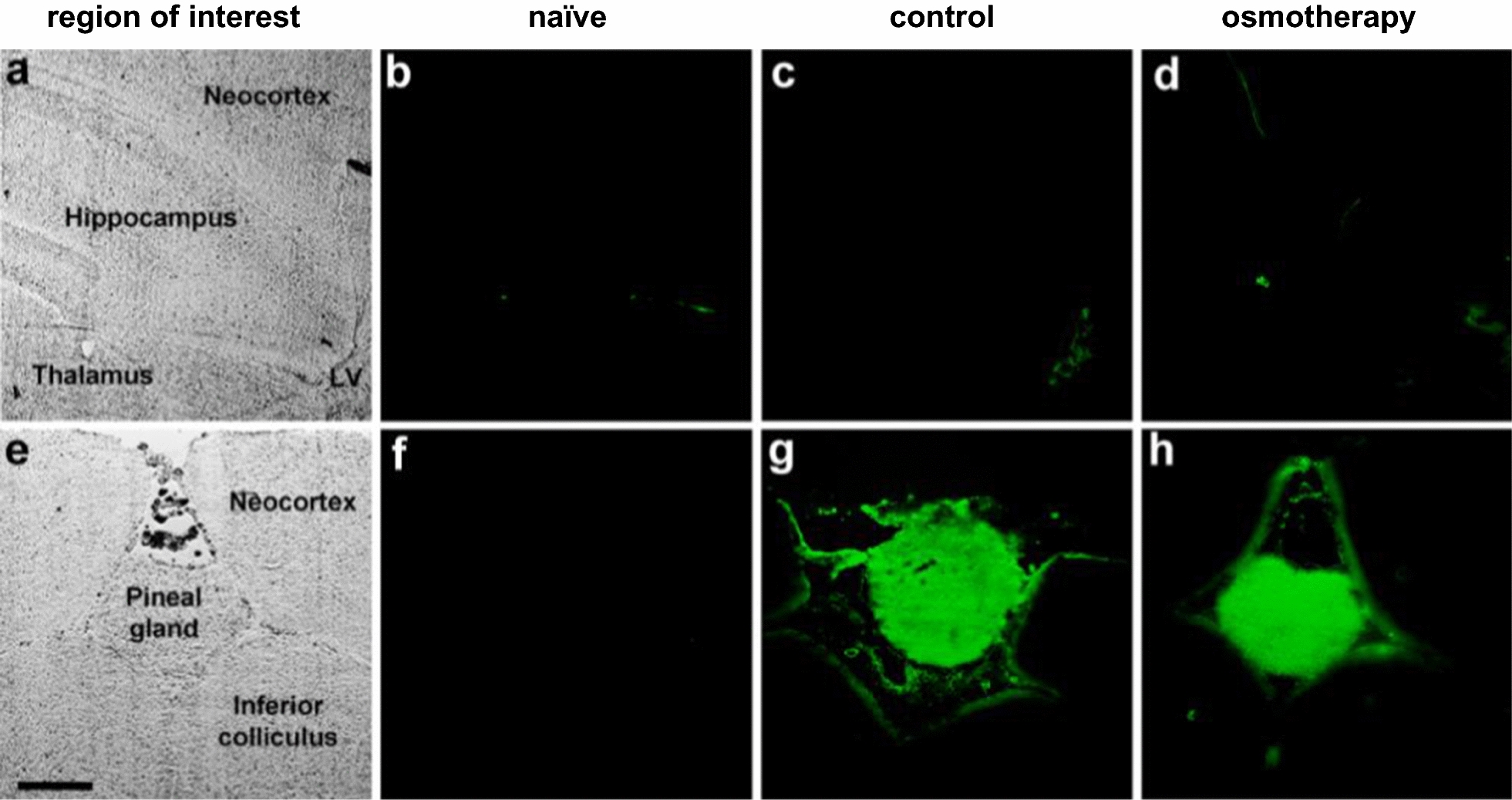
Osmotherapy does not alter the brain barrier permeability. Na^+^-fluorescein (green fluorescence) was injected into the blood circulation of rats prior to i.p. exposure of isosmolar NaCl (control) or hyperosmolar NaCl (osmotherapy). Naïve rats did not receive Na^+^-fluorescein and were euthanized immediately after anaesthesia induction. **a**, **e** Phase contrast images illustrate structures of the brain regions of interest in transmitted white light. Representative images of Na^+^-fluorescein in **b**–**d** hippocampus, thalamus, neocortex, and the lateral ventricle (LV) and **f**–**h** pineal gland (positive control) of naïve rats, control rats, and osmotherapy-treated rats, n = 3. Scale bar = 500 μm

To assess whether Na^+^ and Cl^−^ entered the brain through a possible breach in the brain barriers in response to osmotherapy, we delivered Na^+^-fluorescein i.v. 5 min prior to osmotherapy treatment (as above). Histological analysis of coronal brain sections (Fig. 5a) from the control rats revealed a weak background fluorescent signal in the brain parenchyma, as illustrated before [23], near-absence of Na^+^-fluorescein in the neocortex, hippocampus, and thalamus, and minor staining in the lateral ventricle [23] (from choroid plexus with fenestrated blood capillaries), n = 3, Fig. 5c. Notably, the observed staining pattern was unaltered by osmotherapy treatment as illustrated in representative images of the neocortex, hippocampus, thalamus, and lateral ventricle, while no fluorescence was detected in naïve rats, which did not receive Na^+^-fluorescein (n = 3, Fig. 5b–d). The pineal gland (Fig. 5e) served as a positive control due to the lack of BBB in this brain structure. Hence, Na^+^-fluorescein was detected in the pineal gland of control rats and osmotherapy-treated rats, while no fluorescence was observed in the pineal gland of naïve rats, which did not receive Na^+^-fluorescein (n = 3, Fig. 5f–h). The absence of osmotherapy-induced penetration of Na^+^-fluorescein into the brain indicates that the integrity of the BBB and BCSFB remained intact during the applied osmotherapy treatment.

## Discussion

We have demonstrated in rats that following osmotherapy (~ 50 mOsm increase in plasma osmolarity), water is osmotically extracted from the brain, although to a lesser extent than can be predicted from theoretical calculations. The reduced osmotic extraction was assigned predominantly to brain Na^+^ and Cl^−^ accumulation (6–15% for Na^+^ and 22–38% for Cl^−^) and to a minor extent, if any, brain K^+^ accumulation (up to 3% increase) as a function of increased plasma osmolarity, in agreement with an earlier report [14]. Notably, it is not simply the ion *concentration* that increases with the systemic hyperosmolarity but the actual ion *content*. These findings indicate that specific volume regulatory transporting mechanisms are activated in response to and/or as a consequence of increased plasma osmolarity. Employment of NaCl as the osmotic agent contributed to an increased Na^+^ and Cl^−^ concentration in the plasma, which, in itself, could affect the brain electrolyte content. However, we observed that mannitol-mediated osmotherapy of identical magnitude and delivered volume led to similar effects on the brain electrolyte/water content [14], indicating that plasma hyperosmolarity, and not the increased plasma Na^+^ and Cl^−^ concentrations, causes the brain electrolyte accumulation. Osmotic extraction of cerebral fluid was slightly more effective with NaCl as the osmotic agent, rather than mannitol, even though the cerebral accumulation of Na^+^ was significantly higher in rats treated with NaCl. The reduced osmotic fluid extraction (and thus osmolyte increase) observed with mannitol as the osmotic agent may instead be explained by an unknown but substantial influx of other osmolytes, e.g. mannitol itself, which has previously been detected in the rat brain following mannitol-induced elevation in the plasma osmolarity [14]. With the similar Cl^−^ accumulation obtained with both NaCl and mannitol as the osmotic agent, one may, however, from the principle of electroneutrality, expect accumulation of another cationic electrolyte (or reduced retention of a different anion). Taken together, our findings indicate that the osmotherapy-induced rebound response may be regulated differently depending on the osmotic agent applied, although overlapping mechanisms, such as the observed gain of brain Na^+^ and Cl^−^, clearly exist.

According to theoretical considerations based on reflection coefficients of both osmotic agents, i.e. the relative impermeability across the BBB, NaCl treatment has been predicted to induce a larger osmotic response than mannitol [27], as confirmed by our findings. While previous findings demonstrated that NaCl was superior with regard to initial reduction of the ICP, maintenance of a lowered ICP [28, 29], and an increased cerebral water loss [29] in experimental animal models of brain injuries, other researchers observed an equal efficiency of NaCl or mannitol as the osmotic agent [30, 31], or a higher efficiency with mannitol in healthy animals [32]. Two of the latter observations may, however, be influenced by the unequal end plasma osmolarity induced by either osmotic agent [30, 32], which essentially prevents a comparative analysis. A line of clinical trials, mainly performed on patients with traumatic brain injury, reported that osmotherapy using NaCl solutions with additives (e.g. dextran, lactate, or hydroxyethyl starch solutions) [33–35] or NaCl alone [36] more effectively lowered the ICP compared with mannitol. While these reports support the findings from our animal experiments, two other clinical trials found an equal efficacy of the two osmotic agents on the ICP [37, 38]. However, a direct comparison between the few head-to-head studies carried out is challenged by the varying treatment strategies; (i) continuous or bolus injections, (ii) different doses/volumes of the osmotic agent, and (iii) different time windows, which altogether resulted in variable plasma osmolarities. In addition, diverse patient populations and outcome measurements [39] further hamper the comparison between clinical trials. It is, therefore, still questionable which osmotic agent is superior [1, 40] and animal/clinical studies, which allow direct comparison, are warranted. Mannitol remains the recommended standard osmotic agent for treatment of patients with severe head injury (Level II evidence), whereas hyperosmolar NaCl is recommended for children (Level III evidence) [41]. The choice of osmotic agent may, however, rather be based on side-effect profiles of the osmotic agents and how those will affect the clinical situation (comorbidities, age) [1].

Neither the signaling cascades, nor the molecular transport mechanisms, that couple systemic plasma hyperosmolarity to brain electrolyte accumulation have been identified. In the present study, we therefore introduced a mixture of inhibitors targeting ion-transporting proteins expressed in the BBB capillary endothelium and/or the choroid plexus epithelium, and determined their effect on osmotherapy-induced brain ion accumulation. While amiloride and methazolamide may target abluminal ion-transporting mechanisms [21, 42], we expect insignificant bumetanide interaction at the abluminal membrane of the capillaries forming the BBB because of its poor BBB permeability [43, 44]. We failed to detect evidence in favor of NKCC1, NHE1, ENaC or carbonic anhydrase (indirectly targeting the bicarbonate transporters) located at the BBB endothelium or in choroid plexus participating in this brain volume regulation. Hence, we were unable to reproduce a previously reported reduction of hyperosmotic plasma-induced brain water extraction by methazolamide [14]. The reasons for this discrepancy are unclear, although the previous study employed a very high dose of methazolamide, which was delivered i.p. instead of i.v. as in the present study. We cannot rule out that the inhibitor concentrations applied in this study were not sufficient for effective blockage of the target proteins, even though a procedure with triple doses was incorporated to enhance inhibitor efficiency. The free unbound inhibitor concentration may, however, be significantly reduced by potential binding of inhibitors to plasma proteins, as shown for bumetanide [45]. We recently found that hyperosmotic conditions enhanced the activity of abluminal Na^+^/K^+^-ATPase in endothelial cells, which were co-cultured with astrocytes in an in vitro BBB model [46], indicating that this transport mechanism may counteract osmotic extraction from the brain by cerebral accumulation of Na^+^ in response to a hyperosmotic challenge. With the damaging effect of pump inhibition, it is, however, not simple to verify this finding by currently available techniques in animal models in vivo: a direct effect of Na^+^/K^+^-ATPase inhibition is difficult to deduce, due to disruption of electrochemical gradients controlling secondary active and passive transporting mechanisms. The Na^+^/K^+^-ATPase expressed at the CSF-facing membrane of the choroid plexus could also be a potential candidate in brain volume regulation upon osmotherapy, since the Na^+^/K^+^-ATPase may contribute to CSF production [47], in addition to the recently reported significant contribution of NKCC1 in murine CSF production [48]. To this end, it is important to note that the wet-dry technique, employed to determine brain water content, favors parenchymal water content over CSF, as the major part of CSF is lost in the brain isolation process. If the ion-transporting mechanisms were to regulate the CSF production per se, and the equilibrium rate between CSF and brain interstitial fluid is slow, such regulatory functions could well be missed by this experimental design.

While the ion-transporting mechanisms (NKCC1, NHE1, NBCs, and ENaC) at the BBB capillary endothelium and choroid plexus epithelium were shown not to be involved in the osmotherapy-mediated translocation of Na^+^ and Cl^−^ from the blood into the rat brain under our experimental conditions, Na^+^ and Cl^−^ could instead enter the brain via paracellular transport routes, which may become available with hyperosmolar plasma. However, we demonstrated that the two major brain barriers, i.e. the BBB and BCSFB, appeared to remain intact upon osmotherapy, as we detected no changes in cerebral Na^+^-fluorescein accumulation whether or not the animals had been exposed to osmotherapy. Notably, we cannot exclude that Na^+^ and Cl^−^, which are of a smaller molecular weight (22.99 Da and 35.45 Da) than Na^+^-fluorescein (376.27 Da), can cross the brain barriers via a paracellular route *provided* that the given hyperosmotic challenge promoted an increase in the permeability of the brain barriers towards smaller permeants, while excluding the fluorescent dye. However, a previous study showed that a change in barrier function, corresponding to BBB opening towards mannitol and Na^+^, occurred only with hyperosmotic challenges rendering the plasma osmolarity > 385 mOsm [49]. An alternative manner of accumulating brain electrolytes during conditions of elevated plasma osmolarity could be via increased bulk flow of CSF into the brain interstitial fluid [50] or via a potential regulation of fluid drainage at arachnoid granulations [51], dural lymphatic vessels [52, 53], and/or at glymphatic paravascular drainage routes [54]. Parenchymal cell volume regulation may, in addition, indirectly affect electrolyte movement across the brain barriers.

The present experimental protocol was designed to quantitatively resolve the *direct* consequences of increased plasma osmolarity (mimicked osmotherapy) on brain water and ion accumulation (hence the choice of nephrectomized animals, in which the inflicted change in plasma osmolarity could be tightly controlled). In various severities of stroke-induced brain edema in animal models, one may well expect altered BBB integrity (in the afflicted area) and potentially even altered expression/activity of membrane transporters in the BBB capillary endothelium. Such stroke-induced membrane transport responses could potentially affect ion and water accumulation during osmotherapy, and may serve to explain the observed beneficial effect of bumetanide treatment in an animal stroke model [11]. Future studies should therefore address whether the osmotherapy-mediated influx of cerebral Na^+^ and Cl^−^ likewise contribute to the rebound response in animal models of stroke-induced cerebral edema.

## Conclusions

While osmotherapy immediately lowers the ICP of patients with cerebral edema, a delayed rebound response can limit or even reverse the otherwise effective drainage. We here demonstrated that the mammalian brain loses less water than predicted from osmotically obliged water extraction when exposed to hyperosmolar plasma; osmotherapy. This volume regulatory mechanism, the rebound effect, hinges on initiation of brain ion accumulation predominantly in the form of Na^+^ and Cl^−^. We propose that the brain ion accumulation occurs via transcellular pathways, one of which may well be hyperosmolar-induced abluminal Na^+^/K^+^-ATPase activity [46], rather than due to a hyperosmolar-induced breach in the brain barriers. In the absence of identified luminal transport mechanisms, altered bulk flow (CSF-to-parenchyma flow) or drainage ((g)lymphatic pathways) may well contribute to osmolarity-induced brain electrolyte accumulation. The transport mechanisms proposed to promote osmotherapy-induced brain ion accumulation remain unresolved, since we found no evidence of NKCC1, NHE1, ENaC, and NBCs appearing amongst these under our experimental conditions in healthy non-edematous rats. Future identification of such ion-transporting mechanisms might provide a useful therapeutic target for pharmacological prevention of the rebound effect during osmotherapy in patients experiencing brain edema.

## Abbreviations

aCSF: artificial CSF
ANOVA: analysis of variance
BBB: blood–brain barrier
BCSFB: blood–CSF barrier
CSF: cerebrospinal fluid
ENaC: amiloride-sensitive Na^+^ channel
ICP: intracranial pressure
i.p.: intraperitoneal
i.v.: intravenous
NBCs: Na^+^-coupled bicarbonate transporters
NHE1: Na^+^–H^+^ anti-porter 1
NKCC1: Na^+^–K^+^–2Cl^−^ co-transporter 1
RT: room temperature
SEM: standard error of mean
